# Supplementary material for: Quantification of absolute labeling efficiency at the single-protein level
Source: Nat Methods. 2024 Apr 24;21(9):1702–7. doi: 10.1038/s41592-024-02242-5 (PMC11399078; doi:10.1038/s41592-024-02242-5)
Supplement: Supplementary file 1 — Supplementary Tables 1–10. [file 41592_2024_2242_MOESM1_ESM.pdf]

nature methods

Article https://doi.org/10.1038/s41592-024-02242-5

Quantification of absolute labeling efficiency at the single-protein level

In the format provided by the authors and unedited

Supplementary Information

Supplementary Table 1 | Labeling efficiency – Tag-proteins

Target	Binder	Clone	Terminus	Total area (μm^{-2})	Target density (μm^{-2})	Labeling efficiency (%)	Target-Reference offset (nm)	Cells (n)	Repeats (n)
CD86 - mEGFP	Nanobody	1H1	C	354.0 ± 57.4	48.8 ± 33.7	47.3 ± 2.7	10.3 ± 0.7	6	3
CD86 - mEGFP	Nanobody	1B2	C	547.5 ± 77.4	22.3 ± 8.3	43.8 ± 2.0	10.3 ± 0.5	5	2
CD86 - ALFA-tag	Nanobody	1G5	C	525.4 ± 130.5	29.6 ± 17.0	42.0 ± 5.1	11.8 ± 0.3	6	3
CD86 - mEGFP	Nanobody	1H1<br>1B2	C	331.8 ± 0.0	28.9 ± 15.9	61.8 ± 5.4	13.5 ± 1.7	5	2
CD86 – ALFA-tag-mEGFP	Nanobody	1H1 (GFP)<br>1B2 (GFP)<br>1G5 (Alfa)	C	337.4 ± 68.0	19.5 ± 8.4	76.2 ± 8.4	15.0 ± 0.1	6	2
CD86 - mCherry	Nanobody	2B12	C	360.3 ± 73.0	47.1 ± 33.7	19.7 ± 2.5	11.3 ± 0.5	7	3
CD86 - TagRFP	Nanobody	1H7	C	404.8 ± 88.2	26.0 ± 15.2	41.4 ± 3.4	11.8 ± 0.4	5	2
CD86 - mEOS2	Nanobody	1E8	C	391.6 ± 115.3	13.3 ± 8.0	5.8 ± 1.3	11.8 ± 0.4	5	2
CD86 - mNeonGreen	Nanobody	1E2	C	388.7 ± 105.0	40.3 ± 30.4	35.7 ± 4.1	11.9 ± 0.3	7	3
CD86 - SPOT-tag	Nanobody	bivBC2	C	245.6 ± 56.7	15.0 ± 5.8	10.6 ± 1.8	19.0 ± 1.2	5	2
CD86 - mEGFP	Nanobody	1H1	N	287.6 ± 74.5	57.3 ± 33.3	45.2 ± 4.8	10.0 ± 0.0	6	3
CD70 - mEGFP	Nanobody	1H1	N	259.9 ± 39.1	9.3 ± 4.5	44.3 ± 5.1	11.7 ± 0.4	6	3
CD70 - mEGFP	Nanobody	1H1	C	331.8 ± 0.0	26.9 ± 17.3	45.6 ± 6.5	10.7 ± 0.4	7	3
Nup96-mEGFP	Nanobody	1H1	C	---	---	44.7 ± 0.5	---	2	2

Supplementary Table 2 | Labeling efficiency – Antibodies

Target	Antibody conjugation	Total area (μm^{-2})	Target density (μm^{-2})	Labeling efficiency (%)	Target-Reference offset (nm)	Cells (n)	Repeats (n)
PD-L1	TransGlutaminase	412.9 ± 102.9	62.0 ± 37.2	49.8 ± 14.2	9.4 ± 0.5	9	3
PD-L1	GlyCLICK	534.9 ± 98.3	31.2 ± 5.8	41.3 ± 4.2	9.7 ± 0.4	7	3
CD80	TransGlutaminase	327.1 ± 9.3	15.4 ± 6.7	40.5 ± 6.6	8.7 ± 1.0	7	3
CD80	GlyCLICK	316.6 ± 78.1	32.2 ± 30.0	5.9 ± 3.7	9.6 ± 0.3	10	3
CD86	TransGlutaminase	322.4 ± 58.0	18.2 ± 3.6	39.5 ± 8.3	8.0 ± 1.4	7	3
CD86	GlyCLICK	298.7 ± 71.3	25.7 ± 8.3	34.2 ± 12.7	7.8 ± 0.4	5	3

Supplementary Table 3 | Labeling efficiency – EGFR binders

Target	Binder	Clone	Total area (µm^{-2})	Target density (µm^{-2})	Labeling efficiency (%)	Target-Reference offset (nm)	Cells (n)	Repeats (n)
EGFR	Affibody	AB95116	260.7 ± 26.3	30.9 ± 7.3	17.0 ± 6.8	15.1 ± 0.5	7	3
EGFR	Nanobody	1H1<br>1B2	296.5 ± 91.7	38.6 ± 12.8	62.5 ± 4.3	5.1 ± 0.2	6	3
EGFR	Antibody + 2nd nanobody	199.12 + kLC 1A23	301.7 ± 50.0	38.2 ± 10.6	39.5 ± 5.8	14.6 ± 0.4	11	3
EGFR	Antibody + 2nd nanobody	D38B1 + IgG 10E10	354.0 ± 104.2	24.0 ± 8.5	29.8 ± 6.1	12.8 ± 1.1	12	3

Supplementary Table 4 | EGFR oligomerization

Target	Binder	Clone	Condition	Total area (µm^{-2})	Target density (µm^{-2})	Labeling efficiency (%)	Target-Reference offset (nm)	Monomers (%) – LE-corrected	Dimers (%) – LE-corrected	Monomers (%)	Dimers (%)	Cells (n)	Repeats (n)
EGFR (mEGFP)	Nanobody	1H1<br>1B2	Non-stimulated	276.5 ± 89.9	58.6 ± 20.8	62.2 ± 4.8	5.2 ± 0.3	90.5 ± 4.1	9.5 ± 4.1	93.7 ± 2.6	6.3 ± 2.6	6	3
EGFR (mEGFP)	Nanobody	1H1<br>1B2	EGF-stimulated	264.6 ± 76.2	129 ± 82	59.0 ± 3.6	5.1 ± 0.2	49.3 ± 8.0	50.7 ± 8.0	67.9 ± 6.7	32.1 ± 6.7	9	3
EGFR	Antibody + 2nd nanobody	199.12 + kLC 1A23	Non-stimulated	276.5 ± 89.9	38.8 ± 13.7	43.5 ± 7.9	14.2 ± 0.6	93.7 ± 3.4	6.3 ± 3.4	98.7 ± 1.2	1.3 ± 1.2	6	3
EGFR	Antibody + 2nd nanobody	199.12 + kLC 1A23	EGF-stimulated	264.6 ± 76.2	87.6 ± 46.0	49.3 ± 4.0	12.0 ± 1.3	51.9 ± 8.5	48.1 ± 8.5	78.7 ± 3.4	21.3 ± 3.4	9	3
EGFR	Antibody + 2nd nanobody	D38B1 + IgG 10E10	Non-stimulated	276.5 ± 89.9	28.6 ± 12.6	36.3 ± 8.1	14.2 ± 0.6	94.5 ± 2.7	5.5 ± 2.7	100.0 ± 0.0	0.0 ± 0.0	6	3
EGFR	Antibody + 2nd nanobody	D38B1 + IgG 10E10	EGF-stimulated	264.6 ± 76.2	38.5 ± 21.6	35.4 ± 8.6	13.6 ± 0.8	49.1 ± 7.4	50.9 ± 7.4	86.6 ± 4.0	13.4 ± 4.0	9	3

Supplementary Table 5 | Docking sequences

Docking strand name	Sequence
5xR1	TCCTCCTCCTCCTCCTCCT
7xR3	CTCTCTCTCTCTCTCTC
7xR4	ACACACACACACACACA
5xR6	CAACAACAACAACAACAAC

Supplementary Table 6 | Acquisition parameters for binder specificity experiments

Cell	Target	Binder	Clone	Imager-Sequence	Exposure (ms)	Frames (n)	Power (at objective, mW)	Imager concentration (pM)	Cluster radius xy	Cluster N_{min}
CHO-K1	mEGFP	Nanobody	1H1	R3	75	30 000	25	500	10	10
CHO-K1	mEGFP	Nanobody	1B2	R3	75	30 000	25	500	10	10
CHO-K1	ALFA-tag	Nanobody	1G5	R3	75	30 000	25	500	10	10
CHO-K1	mEGFP	Nanobody	1H1<br>1B2	R3	75	30 000	25	500	10	10
CHO-K1	mEGFP ALFA-tag	Nanobody	1H1<br>1B2<br>1G5	R3	75	30 000	25	500	10	10
CHO-K1	mCherry	Nanobody	2B12	R3	75	30 000	25	500	10	10
CHO-K1	TagRFP	Nanobody	1H7	R3	75	30 000	25	500	10	10
CHO-K1	mEOS2	Nanobody	1E8	R3	75	30 000	25	500	10	10
CHO-K1	mNeonGreen	Nanobody	1E2	R3	75	30 000	25	500	10	10
CHO-K1	SPOT-tag	Nanobody	bivBC2	R3	75	30 000	25	500	10	10
CHO-K1	mEGFP	Nanobody	1H1	R3	75	30 000	25	500	10	10
CHO-K1	PD-L1	Antibody	10F.9G2	R4	75	30 000	25	500	10	10
CHO-K1	CD80	Antibody	16-10A1	R3	75	30 000	25	500	10	10
CHO-K1	CD86	Antibody	GL-1	R1	75	30 000	25	500	10	10
CHO-K1	EGFR	Affibody	AB95116	R3	75	30 000	25	500	10	10
CHO-K1	mEGFP	Nanobody	1H1<br>1B2	R3	75	30 000	25	500	10	10
CHO-K1	EGFR	Antibody + 2nd Nanobody	199.12<br>+ kLC 1A23	R3	75	30 000	25	500	10	10
CHO-K1	EGFR	Antibody + 2nd Nanobody	D38B1 + IgG 10E10	R3	75	30 000	25	500	10	10

Supplementary Table 7 | Acquisition parameters for labeling efficiency experiments

Cell	Construct	Target	Binder	Clone	Imager-Sequence	Reference	Binder	Clone	Imager-Sequence	Exposure (ms)	Frames (n)	Power (at objective, mW)	Imager conc. (pM)	Cluster radius xy	Cluster N_{min}
BSC1	ALFA-CD86-mEGFP	mEGFP	Nanobody	1H1	R3	ALFA-tag	Nanobody	1G5	R4	75	30 000	25	500	10	10
BSC1	mEGFP-CD86-ALFA	mEGFP	Nanobody	1H1	R3	ALFA-tag	Nanobody	1G5	R4	75	30 000	25	500	10	10
U2OS	Nup96-mEGFP	mEGFP	Nanobody	1H1	R3	---	---	---	---	150	30 000	18	500	10	10
BSC1	ALFA-CD86-mEGFP	mEGFP	Nanobody	1B2	R3	ALFA-tag	Nanobody	1G5	R4	75	30 000	25	500	10	10

BSC1	mEGFP-CD86-ALFA-tag	ALFA-tag	Nanobody	1G5	R3	mEGFP	Nanobody	1H1	R4	75	30 000	25	500	10	10
BSC1	ALFA-CD86-mEGFP	mEGFP	Nanobody	1H1 1B2	R3	ALFA-tag	Nanobody	1G5	R4	75	30 000	25	500	10	10
BSC1	ALFA-CD86-mEGFP	mEGFP ALFA-tag	Nanobody	1H1 1B2 1G5	R3	CD86	Antibody	GL-1	R1	75	30 000	25	500	10	10
BSC1	ALFA-CD86-mEGFP	mCherry	Nanobody	2B12	R3	ALFA-tag	Nanobody	1G5	R4	75	30 000	25	500	10	10
BSC1	ALFA-CD86-mEGFP	TagRFP	Nanobody	1H7	R3	ALFA-tag	Nanobody	1G5	R4	75	30 000	25	500	10	10
BSC1	ALFA-CD86-mEGFP	mEOS2	Nanobody	1E8	R3	ALFA-tag	Nanobody	1G5	R4	75	30 000	25	500	10	10
BSC1	ALFA-CD86-mEGFP	mNeonGreen	Nanobody	1E2	R3	ALFA-tag	Nanobody	1G5	R4	75	30 000	25	500	10	10
BSC1	ALFA-CD86-mEGFP	SPOT-tag	Nanobody	bivBC2	R3	ALFA-tag	Nanobody	1G5	R4	75	30 000	25	500	10	10
BSC1	ALFA-CD70-mEGFP	mEGFP	Nanobody	1H1	R3	ALFA-tag	Nanobody	1G5	R4	75	30 000	25	500	10	10
BSC1	mEGFP-CD70-ALFA	mEGFP	Nanobody	1H1	R3	ALFA-tag	Nanobody	1G5	R4	75	30 000	25	500	10	10
CHO-K1	PD-L1-ALFA-mEGFP	PD-L1	Antibody	10F.9G2	R4	ALFA-tag	Nanobody	1G5	R3	75	30 000	25	500	10	10
CHO-K1	CD80-ALFA-mEGFP	CD80	Antibody	16-10A1	R3	ALFA-tag	Nanobody	1G5	R4	75	30 000	25	500	10	10
CHO-K1	CD86-ALFA-mEGFP	CD86	Antibody	GL-1	R1	ALFA-tag	Nanobody	1G5	R4	75	30 000	25	500	10	10
CHO-K1	EGFR-ALFA-mEGFP	EGFR	Affibody	AB95116	R3	ALFA-tag	Nanobody	1G5	R4	75	30 000	25	500	10	10
CHO-K1	EGFR-ALFA-mEGFP	mEGFP	Nanobody	1H1 1B2	R3	ALFA-tag	Nanobody	1G5	R4	75	30 000	25	500	10	10
CHO-K1	EGFR-ALFA-mEGFP	EGFR	Antibody + 2nd Nanobody	199.12 + kLC 1A23	R3	ALFA-tag	Nanobody	1G5	R4	75	30 000	25	500	10	10

CHO-K1	EGFR-ALFA-mEGFP	EGFR	Antibody + 2nd Nanobody	D38B1 + IgG 10E10	R3	ALFA-tag	Nanobody	1G5	R4	75	30 000	25	500	10	10

Supplementary Table 8 | Acquisition parameters for EGFR experiments

Cell	Construct	Target	Binder	Clone	Imager-Sequence	Reference	Binder	Clone	Imager-Sequence	Exposure (ms)	Frames TARGET / REFERENCE(n)	Power (at objective, mW)	Imager concentration -TARGET / REFERENCE (pM)	Cluster radius xy	Cluster N_{min}
CHO-K1	EGFR-ALFA-mEGFP	mEGFP	Nanobody	1H1 1B2	R3	ALFA-tag	Nanobody	1G5	R4	75	50 000 / 30 000	25	250 / 500	10	10
CHO-K1	EGFR-ALFA-mEGFP	EGFR	Antibody + 2nd Nanobody	199.12 + kLC 1A23	R1	ALFA-tag	Nanobody	1G5	R4	75	50 000 / 30 000	25	250 / 500	10	10
CHO-K1	EGFR-ALFA-mEGFP	EGFR	Antibody + 2nd Nanobody	D38B1 + IgG 10E10	R6	ALFA-tag	Nanobody	1G5	R4	75	50 000 / 30 000	25	250 / 500	10	10

Supplementary Table 9 | Imager sequences

Imager name	Sequence	3'-mod
R1	AGGAGGA	Cy3b
R3	AGAGAGA	Cy3b
R4	GTGTGT	Cy3b
R6	TGTTGTT	Cy3b

Supplementary Table 10 | Binder specificity

Target	Binder	Clone	Total area (μm^{-2})	Binder density (μm^{-2})	Cells (n)	Repeats (n)
mEGFP	Nanobody	1H1	331.8 ± 0.0	0.19 ± 0.05	5	2
mEGFP	Nanobody	1B2	331.8 ± 0.0	0.23 ± 0.05	5	2
ALFA-tag	Nanobody	1G5	404.8 ± 31.9	0.06 ± 0.05	5	2
mCherry	Nanobody	2B12	331.8 ± 0.0	0.61 ± 0.56	5	2
TagRFP	Nanobody	1H7	331.8 ± 0.0	0.41 ± 0.15	5	2
mEOS2	Nanobody	1E8	331.8 ± 0.0	0.35 ± 0.09	5	2
mNeonGreen	Nanobody	1E2	331.8 ± 0.0	0.30 ± 0.09	5	2
SPOT-tag	Nanobody	bivBC2	331.8 ± 0.0	0.46 ± 0.12	5	2
PD-L1	Antibody (TransGlutaminase)	10F.9G2	331.8 ± 0.0	0.31 ± 0.02	5	2
PD-L1	Antibody (GlyCLICK)	10F.9G2	331.8 ± 0.0	0.28 ± 0.10	5	2
CD80	Antibody (TransGlutaminase)	16-10A1	338.5 ± 13.0	0.21 ± 0.05	5	2
CD80	Antibody (GlyCLICK)	16-10A1	371.7 ± 31.9	0.39 ± 0.09	5	2
CD86	Antibody (TransGlutaminase)	GL-1	351.7 ± 26.0	0.18 ± 0.03	5	2
CD86	Antibody (GlyCLICK)	GL-1	345.1 ± 26.0	0.37 ± 0.05	5	2
EGFR	Affibody	AB95116	348.4 ± 32.5	0.62 ± 0.21	8	2
EGFR	Antibody + 2nd Nanobody	199.12 + kLC 1A23	331.8 ± 0.0	0.37 ± 0.08	5	2
EGFR	Antibody + 2nd Nanobody	D38B1 + IgG 10E10	331.8 ± 0.0	0.38 ± 0.11	5	2
