# Supplementary material for: Quantification of absolute labeling efficiency at the single-protein level
Source: Nat Methods. 2024 Apr 24;21(9):1702–7. doi: 10.1038/s41592-024-02242-5 (PMC11399078; doi:10.1038/s41592-024-02242-5)
Supplement: Supplementary file 2 — Reporting Summary [file 41592_2024_2242_MOESM2_ESM.pdf]

nature portfolio

Corresponding author(s): Ralf Jungmann

Last updated by author(s): Feb 26, 2024

Reporting Summary

Nature Portfolio wishes to improve the reproducibility of the work that we publish. This form provides structure for consistency and transparency in reporting. For further information on Nature Portfolio policies, see our Editorial Policies and the Editorial Policy Checklist.

Statistics

For all statistical analyses, confirm that the following items are present in the figure legend, table legend, main text, or Methods section.

n/a	Confirmed	
☐	☒	The exact sample size (*n*) for each experimental group/condition, given as a discrete number and unit of measurement
☐	☒	A statement on whether measurements were taken from distinct samples or whether the same sample was measured repeatedly
☐	☒	The statistical test(s) used AND whether they are one- or two-sided<br>*Only common tests should be described solely by name; describe more complex techniques in the Methods section.*
☒	☐	A description of all covariates tested
☒	☐	A description of any assumptions or corrections, such as tests of normality and adjustment for multiple comparisons
☐	☒	A full description of the statistical parameters including central tendency (e.g. means) or other basic estimates (e.g. regression coefficient) AND variation (e.g. standard deviation) or associated estimates of uncertainty (e.g. confidence intervals)
☐	☒	For null hypothesis testing, the test statistic (e.g. *F*, *t*, *r*) with confidence intervals, effect sizes, degrees of freedom and *P* value noted<br>*Give P values as exact values whenever suitable.*
☒	☐	For Bayesian analysis, information on the choice of priors and Markov chain Monte Carlo settings
☒	☐	For hierarchical and complex designs, identification of the appropriate level for tests and full reporting of outcomes
☒	☐	Estimates of effect sizes (e.g. Cohen's *d*, Pearson's *r*), indicating how they were calculated

Our web collection on statistics for biologists contains articles on many of the points above.

Software and code

Policy information about availability of computer code

Data collection	Raw microscopy data was acquired using µManager (Version 2.0-gamma) (Edelstein, A., Amodaj, N., Hoover, K., Vale, R. & Stuurman, N. Curr. Protoc. Mol. Biol. 14.20 (2010))
Data analysis	All data were analyzed using the open source software Picasso (Versions 0.6.0 - 0.6.5) (Schnitzbauer, J., Strauss, M. T., Schlichthaerle, T., Schueder, F., & Jungmann, R. (2017). Super-resolution microscopy with DNA-PAINT. Nature Protocols, 12(6), 1198–1228. http://doi.org/10.1038/nprot.2017.024). Latest Version can be found on GitHub: https://github.com/jungmannlab/picasso)

For manuscripts utilizing custom algorithms or software that are central to the research but not yet described in published literature, software must be made available to editors and reviewers. We strongly encourage code deposition in a community repository (e.g. GitHub). See the Nature Portfolio guidelines for submitting code & software for further information.

Data

Policy information about availability of data

All manuscripts must include a data availability statement. This statement should provide the following information, where applicable:

- Accession codes, unique identifiers, or web links for publicly available datasets
- A description of any restrictions on data availability
- For clinical datasets or third party data, please ensure that the statement adheres to our policy

All raw data is available upon reasonable request from the authors.

Human research participants

Policy information about studies involving human research participants and Sex and Gender in Research.

Reporting on sex and gender	n/a
Population characteristics	n/a
Recruitment	n/a
Ethics oversight	n/a

Note that full information on the approval of the study protocol must also be provided in the manuscript.

Field-specific reporting

Please select the one below that is the best fit for your research. If you are not sure, read the appropriate sections before making your selection.

☒ Life sciences ☐ Behavioural & social sciences ☐ Ecological, evolutionary & environmental sciences

For a reference copy of the document with all sections, see nature.com/documents/nr-reporting-summary-flat.pdf

Life sciences study design

All studies must disclose on these points even when the disclosure is negative.

Sample size	Sample size n is defined as the number of cells that were recorded and analyzed.
Data exclusions	No data were excluded.
Replication	All replications were successful.
Randomization	N/A, no grouping of experiments or samples were performed.
Blinding	N/A, no grouping of experiments or samples were performed.

Reporting for specific materials, systems and methods

We require information from authors about some types of materials, experimental systems and methods used in many studies. Here, indicate whether each material, system or method listed is relevant to your study. If you are not sure if a list item applies to your research, read the appropriate section before selecting a response.

Materials & experimental systems

n/a	Involved in the study	
☐	☒	Antibodies
☐	☒	Eukaryotic cell lines
☒	☐	Palaeontology and archaeology
☒	☐	Animals and other organisms
☒	☐	Clinical data
☒	☐	Dual use research of concern

Methods

n/a	Involved in the study	
☒	☐	ChIP-seq
☒	☐	Flow cytometry
☒	☐	MRI-based neuroimaging

Antibodies

Antibodies used

1) Anti-PD-L1 Rat Monoclonal, BioLegend (cat: 124302, clone 10F.9G2), dilution 50 nM
2) Anti-CD80 Armenian Hamster Monoclonal, BioLegend (cat: 104702, clone 16-10A1), dilution 50 nM
3) Anti-CD86 Rat Monoclonal, BioLegend (cat: 105002, clone GL-1) , dilution 50 nM
4) Anti-EGFR Mouse Monoclonal, Thermo Fisher Scientific (cat: MA5-13319, clone 199.12), dilution 1 in 100
5) Anti-EGFR Monoclonal, Affibody (Abcam, clone ab81872, unconjugated), dilution 1 in 100
6) Anti-ALFA Nanobody, NanoTag (cat: N1505, clone 1G5), dilution 0.5 - 50 nM
7) Anti-GFP Nanobody, NanoTag (cat: N0305, clone: 1H1), dilution 50 nM
8) Anti-Mouse Ig kappa light chain Nanobody, NanoTag (cat: N1205, clone 1A23), dilution 50 nM

	9) Anti-Rabbit IgG Nanobody, NanoTag (cat: N2405, clone 10E10), dilution 50 nM<br>10) Anti-RFP Nanobody, NanoTag (cat: N0405, clone 2B12), dilution 50 nM<br>11) Anti-TagFP Nanobody, NanoTag (cat: N0505, clone 1H7), dilution 50 nM<br>12) Anti-mEos Nanobody, NanoTag (cat: N3105, clone 1E8), dilution 50 nM<br>13) Anti-mNeonGreen Nanobody, NanoTag (cat: N3205, clone 1E2), dilution 50 nM<br>14) Anti-SPOT Nanobody, ChromoTek (cat: etb, RRID_2827572), dilution 50 nM<br>15) Anti-EGFR, monoclonal rabbit IgG, Cell Signaling, (cat: 4267, clone: D38B1), dilution 1 in 100<br>16) Anti-GFP Nanobody, NanoTag, (custom, clone 1B2, C-terminal cysteine), dilution 50 nM
Validation	All antibodies and nanobodies were validated by the manufacturers using western blot and immunofluorescence.

Eukaryotic cell lines

Policy information about cell lines and Sex and Gender in Research

Cell line source(s)	CHO-K1 (Sigma Aldrich, 85051005-1VL), BSC1 (Creative-Bioarray, CSC-C9342L), U-2 OS-CRISPR-Nup96-mEGFP cells were obtained from the Ellenberg and Ries lab (Reference: https://doi.org/10.1038/s41592-019-0574-9).
Authentication	The cell lines were not authenticated.
Mycoplasma contamination	All cell lines have been tested negative for mycoplasma contamination.
Commonly misidentified lines (See ICLAC register)	No commonly misidentified cell lines were used.
